# Relationship between persistent atrial fibrillation in high-altitude regions and left atrial diameter, red cell distribution width, and NT-ProBNP: a retrospective case-control study

**DOI:** 10.3389/fcvm.2026.1724615

**Published:** 2026-06-30

**Authors:** Jiandong Cao, Wenxing Zhao, Xiaobing Duan, Longxiang Zhao, Xiaofeng Ma

**Affiliations:** 1 Department of Cardiology, Qinghai Province Cardio Cerebrovascular Disease Specialist Hospital, Xining, Qinghai, China; 2 Interventional Centers, Qinghai Province Cardio Cerebrovascular Disease Specialist Hospital, Xining, Qinghai, China; 3 Qinghai University Affiliated Hospital, Xining, Qinghai, China

**Keywords:** left atrial diameter, NT-ProBNP, persistent atrial fibrillation, Qinghai, red blood cell distribution width

## Abstract

**Objective:**

This study aimed to examine the association of left atrial diameter (LAD), red cell distribution width (RDW-CV) and N-terminal pro-B-type natriuretic peptide (NT-proBNP) with persistent atrial fibrillation (AF) in high-altitude.

**Methods:**

In this retrospective study, a total of 4,244 patients diagnosed with persistent AF at Qinghai Province Cardiovascular and Cerebrovascular Disease Specialist Hospital between January 2019 and June 2021 were included in the AF group, and 883 healthy individuals were selected as the control group. Factors associated with AF were evaluated. The AF group was stratified into middle-altitude and high-altitude groups. Comparisons of cardiac structural parameters and hematological indicators were performed across ethnic and altitude groups.

**Results:**

LAD, N-terminal pro-B-type natriuretic peptide (NT-proBNP), and the coefficient of variation of RDW (RDW-CV) were identified as independent factors associated with AF [odds ratio (OR), 95% confidence interval (CI) = 1.014–1.241, 1.002–1.010, 1.282–1.655; *p* < 0.05]. In the middle-altitude group, significant differences were observed among patients of Tibetan, Han, and Hui ethnicity in terms of NT-proBNP (*p* < 0.05). In the high-altitude group, significant differences were found in RDW-CV (*p* < 0.05). Among patients of Hui ethnicity with AF, difference in NT-proBNP was observed between middle- and high-altitude groups (*p* < 0.05).

**Conclusion:**

LAD, NT-proBNP, and RDW-CV were factors associated with AF. LAD has a consistent association on patients with atrial fibrillation across different altitudes and ethnic groups.

## 1 Introduction

Atrial fibrillation (AF) is a common cardiac arrhythmia associated with high mortality and disability rates. By 2021, global incidence reached 47.05 per 100,000 population, prevalence 397.94, mortality 0.49, and disability 48.46 (1). Its high prevalence has resulted in a significant public health concern (1).

The occurrence of AF is associated with multiple risk factors. Echocardiographic parameters, including left ventricular hypertrophy, left atrial enlargement, increased left ventricular end-diastolic diameter (LVEDD), and increased left ventricular wall thickness, have been indicated as risk factors for AF (2, 3). In addition, red cell distribution width (RDW-CV) has been identified as an independent risk factor for AF and has been applied as an indicator in stroke risk assessment among patients with AF (4–6). Hematocrit (HCT) has also been reported to be significantly elevated in patients with chronic persistent AF, contributing to increased whole-blood viscosity and promoting thrombus formation (7).

Qinghai Province is located in the northwest of China, on the eastern part of the Qinghai-Tibet Plateau, with an average elevation of 3,000 m. It has a permanent resident population of 5.95 million, with Han people accounting for 53.02%, Tibetans accounting for 24.44%, and Hui people accounting for 14.52% (8). The high-altitude environment is characterized by low temperature, low pressure, low oxygen, and strong ultraviolet radiation, among which hypoxia is the main feature. Hypoxia leads to changes in the transcription and translation of multiple genes mediated by transcription regulatory factors such as hypoxia-inducible factors, which in turn cause a series of pathophysiological changes such as energy metabolism imbalance, neuroendocrine alteration, fluid imbalance, increased oxidative stress, and vascular dysfunction (9, 10). In an inflammatory and oxidative stress environment, the environment required for red blood cell (RBC) maturation is destroyed by inflammation and oxidative stress, resulting in a large number of immature RBCs in the blood circulation, leading to an increase in red blood cell distribution width (RDW-CV) (11, 12). In addition, inflammation and oxidative stress are involved in the formation of AF, myocardial cell inflammatory infiltration, fibrosis, apoptosis, and even necrosis, atrial enlargement, and tissue fibrosis. Fibrosis disrupts the continuity of fiber bundles, causing local fractures and promoting conduction disturbances in AF (13). Among people residing at a high altitude, red blood cell count, hemoglobin concentration, and HCT often exceed values observed in people residing in lower altitudes due to chronic hypoxia (14). This hematologic profile is characterized by increased cellular concentration, aggregation, and viscosity, which elevates cardiac workload, induces systemic and pulmonary circulatory disturbances, and contributes to cardiac structural remodeling (7).

Qinghai Province is a multiethnic region primarily inhabited by people of Tibetan, Han, and Hui ethnicities. These ethnic groups exhibit differences in lifestyle and adaptive capacity to high altitude hypoxic environments. The interaction between altitude and ethnicity exerts varying effects on AF risk and clinical presentation (15, 16). To better understand the contribution of ethnic adaptation and altitude-related factors to AF, the present study evaluated the associations of LAD, RDW-CV, and NT-proBNP with AF, and analyzes their differences across various altitudes and ethnic groups. The objective was to identify the factors associated with AF among people living high-altitude regions, support evidence-based strategies for prevention and treatment.

## 2 Materials and methods

### 2.1 Study population

This study retrospectively analyzed 4,244 patients with persistent AF who had previously been admitted to our hospital. The diagnostic criteria for AF were based on the 2019 American Heart Association/American College of Cardiology/Heart Rhythm Society focused update of the 2014 Atrial Fibrillation Guideline, defined as AF confirmed by standard 12-lead electrocardiography or Holter monitoring (17).

Patients were consecutively enrolled at the time of hospitalization, either through elective admission or via the emergency department. Indications for hospitalization included pulmonary infection, acute coronary syndrome, acute exacerbation of heart failure, elective surgery, and poorly controlled blood pressure. Patients were admitted to specialized departments, including hypertension, arrhythmia, heart failure, coronary heart disease, geriatrics, and the intensive care unit (ICU). A total of 883 healthy individuals who underwent physical examinations during the same period were recruited as the control group.

### 2.2 Definition

The diagnostic criteria for hypertension followed the 2018 Chinese Guidelines for the Prevention and Treatment of Hypertension, defined as repeated office blood pressure ≥140/90 mmHg, or ambulatory blood pressure monitoring demonstrating 24 h mean blood pressure ≥130/80 mmHg, daytime mean blood pressure ≥135/85 mmHg, or nighttime mean blood pressure ≥120/70 mmHg (18).

The diagnostic criteria for type 2 diabetes mellitus (T2DM) followed the 2017 Chinese Guidelines for the Prevention and Treatment of T2DM, defined as glycated hemoglobin ≥6.5%, fasting plasma glucose ≥7.0 mmol/L, or 2 h plasma glucose ≥11.1 mmol/L during an oral glucose tolerance test (19).

Smoking history was defined as daily smoking of ≥10 cigarettes for a long duration or smoking cessation within the preceding 6 months.

Medium altitude was defined as residence at 1,500–2,500 m, and high altitude as ≥2,500 m.

### 2.3 Data collection

Demographic and clinical data were collected, including sex, ethnicity, residential altitude, age, history of hypertension and blood pressure values, history of diabetes, smoking status, history of cor pulmonale, history of coronary heart disease, and history of surgery. For each patient, data from their first hospitalization were used as baseline enrollment data.

On the second day after admission, 2 mL of fasting peripheral venous blood was collected into tubes containing 2% EDTA dipotassium salt. Red blood cell count (RBC), hemoglobin, HCT, platelet count, mean corpuscular volume, and RDW-CV were measured using a Sysmex XT-1800i automated hematology analyzer.

An additional 4 mL of fasting peripheral venous blood was collected into tubes containing a coagulation-promoting agent, centrifuged at 3,000 r/min for 10 min (centrifugal radius 10 cm), and the serum was separated. Serum total bilirubin, direct bilirubin (DBiL), indirect bilirubin (IBiL), uric acid, homocysteine (Hcy), blood glucose, C-reactive protein (CRP), superoxide dismutase (SOD), and angiotensin-converting enzyme were measured using a Beckman Coulter AU5800 automated biochemical analyzer.

On the second day of admission, echocardiography was performed by experienced physicians using a Vivid I cardiovascular ultrasound system (S5, GE Healthcare, Horten, Norway). Parameters recorded included main pulmonary artery width (MPAW), left atrial diameter (LAD), left ventricular end-diastolic diameter (LVEDD), interventricular septal thickness (IVST), left ventricular end-systolic diameter, left ventricular end-diastolic volume, interventricular septal thickness, right atrial diameter (RAD), right ventricular diameter (RVD), and left ventricular ejection fraction (LVEF).

### 2.4 Statistical analysis

All data were analyzed using SPSS version 20.0 software. Measurement data are expressed as mean ± standard deviation, and categorical data are expressed as percentages. Differences between two groups of measurement data were assessed using the independent-sample *t*-test, and comparisons among multiple groups were performed using one-way analysis of variance. For data not normally distributed, the Kruskal–Wallis test was applied. Differences in categorical data were assessed using the chi-squared (*χ*^2^) test.

Logistic regression analysis was conducted to identify factors associated with the presence of AF. Variables with *p* < 0.10 in univariate analysis, as well as clinically relevant variables (i.e., sex, age, ethnicity, hypertension, type 2 diabetes, smoking, MPAW, LAD, IVST, LVEDD, RAD, RVD, LVEF, NT-proBNP, D-Dimer, WBC, RBC, RDW-CV, DBIL, IBIL, CysC, UA, Hcy, and SOD), were included in the multivariable logistic regression model. Continuous variables (e.g., age, MPAW, LAD, IVST, LVEDD, RAD, RVD, LVEF, NT-proBNP, D-Dimer, WBC, RBC, RDW-CV, DBIL, IBIL, CysC, UA, Hcy, and SOD) were entered as continuous variables, while categorical variables (e.g., sex, ethnicity, hypertension, type 2 diabetes, and smoking) were entered as categorical variables. Adjusted odds ratios (ORs) and 95% confidence intervals (CIs) were calculated.

The diagnostic value of each variable for AF is assessed using the ROC curve, a value of *p* < 0.05 was considered statistically significant.

## 3 Results

### 3.1 Comparison between the AF group and the control group

#### 3.1.1 Baseline characteristics

Baseline characteristics of the patients are presented in Table 1. The proportion of females was significantly higher in the AF group compared with the control group (48.85% vs. 34.43%, *p* < 0.05). Patients with AF were older than those in the control group (*p* < 0.05). The proportion of smokers was also higher in the AF group than in the control group (17.78% vs. 11.89%, *p* < 0.05). The prevalence of hypertension was greater in the AF group (25.09% vs. 17.10%, *p* < 0.05), and the prevalence of diabetes was also higher among patients with AF (13.25% vs. 2.38%, *p* < 0.05). Significant differences in ethnic composition were observed between the two groups.

**Table 1:** Baseline characteristics of patients in the AF and control groups.

Indicator		Control group (*n* = 883)	AF group (*n* = 4,244)	*Z*/*χ*^2^	*p*
Sex	Female	304 (34.43)	2,073 (48.85)	61.095	0.000
	Male	579 (65.57)	2,171 (51.15)		
Age		57 (50.00, 66.00)	69 (61.00, 76.00)	−22.592	0.000
Ethnicity	Han Chinese	528 (59.80)	3,039 (71.61)	67.145	0.000
	Tibetan	156 (17.67)	399 (9.40)		
	Hui	144 (16.31)	547 (12.89)		
	Others	55 (6.23)	259 (6.10)		
Hypertension	No	732 (82.90)	3,179 (74.91)	68.180	0.000
	Yes	151 (17.10)	1,065 (25.09)		
Type 2 diabetes	No	862 (97.62)	3,681 (86.75)	85.630	0.000
	Yes	21 (2.38)	562 (13.25)		
Smoking	No	778 (88.11)	3,485 (82.12)	185.356	0.000
	Yes	105 (11.89)	759 (17.88)		
Altitude	Middle-altitude	550 (62.29)	2,720 (64.09)	0.384	0.535
	High-altitude	333(37.71)	1,524(35.91)		

Categorical variables are expressed as frequency (percentage). Continuous variables are expressed as median (25th percentile, 75th percentile) owing to non-normal distribution.

#### 3.1.2 Echocardiographic and hematological parameters

Comparisons of echocardiographic and hematological indicators are presented in Table 2. Significant differences (*p* < 0.05) were observed between the AF group and the control group in main pulmonary artery diameter, LAD, N-terminal pro-B-type natriuretic peptide (NT-proBNP), and RDW-CV.

**Table 2:** Comparison of echocardiographic and hematological parameters between the AF and control groups.

Indicator	Control group (*n* = 883)	AF group (*n* = 4,244)	*Z*	*p*
MPAW mm	20.000 (19.0, 22.0)	27.000 (25.0, 28.0)	−41.018	0.000
LAD mm	50.000 (50.0, 50.0)	66.000 (64.0, 66.0)	−46.364	0.000
IVST mm	8.000 (8.0, 9.0)	10.000 (10.0, 11.0)	−30.098	0.000
LVEDD mm	46.000 (43.0, 48.0)	50.000 (46.0, 55.0)	−22.200	0.000
RAD mm	42.000 (40.0, 45.0)	58.000 (52.0, 61.0)	−41.666	0.000
RVD mm	21.000 (20.0, 23.0)	24.000 (22.0, 24.0)	−20.668	0.000
LVEF %	62.000 (60.0, 66.0)	51.000 (49.0, 59.0)	−35.467	0.000
NT-proBNP pg/mL	81.200 (40.1, 81.2)	2,555.000 (992.0, 5,101.0)	−44.103	0.000
D-Dimer μg/mL	0.340 (0.1, 0.3)	0.850 (0.5, 1.7)	−35.853	0.000
WBC × 10^9^/L	6.890 (5.5, 8.2)	6.010 (4.9, 7.4)	−10.320	0.000
RBC × 10^9^/L	5.010 (4.6, 5.3)	4.880 (4.4, 5.4)	−4.185	0.000
HCT %	46.000 (43.0, 50.0)	46.000 (42.0, 50.0)	−1.774	0.076
RDW-CV %	43.700 (42.1, 44.3)	49.300 (46.0, 53.5)	−46.794	0.000
DBIL μmol/L	3.500 (2.7, 4.8)	8.800 (5.9, 13.6)	−36.448	0.000
IBIL μmol/L	8.:900 (6.2, 11.2)	11.500 (8.4, 16.0)	−17.370	0.000
UA μmol/L	428.000 (357.0, 513.0)	393.000 (319.0, 492.0)	−6.807	0.000
CysC mg/L	1.460 (1.2, 1.9)	1.290 (1.0, 1.6)	−8.732	0.000
Hcy μmol/L	14.050 (11.1, 17.7)	13.020 (10.0, 16.4)	−7.194	0.000
SOD U/mL	127.000 (115.0, 140.0)	123.000(110.0, 137.0)	−5.462	0.000

AF, atrial fibrillation; MPAW, main pulmonary artery width; LAD, left atrial diameter; IVST, interventricular septal thickness; LVEDD, left ventricular end-diastolic diameter; RAD, right atrial diameter; RVD, right ventricular diameter; LVEF, left ventricular ejection fraction; NT-proBNP, N-terminal pro-brain natriuretic peptide; D-dimer, D-dimer (fibrin degradation product); WBC, white blood cell count; RBC, red blood cell count; HCT, hematocrit; RDW-CV, red cell distribution width; DBIL, direct bilirubin; IBIL, indirect bilirubin; UA, uric acid; CysC, cystatin C; Hcy, homocysteine; SOD, superoxide dismutase. Categorical variables are expressed as frequency (percentage). Continuous variables are expressed as median (25th percentile, 75th percentile) owing to non-normal distribution.

#### 3.1.3 Multivariable analysis

Logistic regression analysis was conducted with the presence of AF as the dependent variable, while age, hypertension, diabetes, smoking history, main pulmonary artery diameter, LAD, interventricular septal thickness, LVEDD, right atrial diameter, right ventricular diameter, LVEF, NT-proBNP, D-dimer, WBC, RBC, RDW-CV, DBiL, IBiL, uric acid, CysC, Hcy, CRP, and SOD were included as independent variables. The results indicated that LAD, NT-proBNP, and RDW-CV were independent factors associated with the presence of AF (*p* < 0.05), as presented in Table 3. For each 1 mm increase in LAD, the risk of AF increased by 1.7% (odds ratio = 1.017). For each 1% increase in RDW-CV, the risk of AF increased by 45.7% (odds ratio = 1.457). The odds ratio for NT-proBNP (1.006) is close to 1. Multicollinearity was assessed using variance inflation factors (VIFs) (Table 4). All variables had VIF values < 5, indicating no significant multicollinearity in the model.

**Table 3:** Multivariable logistic regression analysis identifying independent risk factors for the presence of AF.

Indicator	Regression coefficient	Standard error	*z* value	Wald *χ*^2^	*p*-value	OR value	95% CI for OR
Sex	0.162	0.075	3.658	1.478	0.058	0.896	0.237–11.418
Age	−0.048	0.036	−1.328	1.764	0.184	0.954	0.889–1.023
Ethnicity	0.194	0.093	1.158	4.390	0.196	1.015	0.746–13.117
Hypertension	0.017	0.102	0.169	0.029	0.866	1.017	0.834–1.241
Type 2 diabetes	0.001	1.222	0.001	0.000	0.999	1.001	0.091–10.982
smoking	0.003	1.568	0.002	0.000	0.998	1.003	0.046–21.664
MPAW mm	0.010	0.177	0.057	0.003	0.955	1.010	0.714–1.429
LAD mm	0.017	0.102	0.169	0.029	0.046	1.017	1.014–1.241
IVST mm	0.003	0.323	0.009	0.000	0.992	1.003	0.533–1.889
LVEDD mm	−0.016	0.014	−1.181	1.394	0.238	0.984	0.958–1.011
RAD mm	0.000	0.093	0.004	0.000	0.997	1.000	0.833–1.202
RVD mm	−0.035	0.193	−0.182	0.033	0.856	0.965	0.661–1.410
LVEF %	−0.193	0.110	−1.764	3.113	0.078	0.824	0.665–1.022
NT-proBNP pg/mL	0.006	0.002	3.112	9.683	0.002	1.006	1.002–1.010
D-Dimer μg/mL	0.005	0.255	0.020	0.000	0.984	1.005	0.610–1.656
WBC × 10^9^/L	−0.028	0.270	−0.103	0.011	0.918	0.973	0.573–1.650
RBC × 10^9^/L	0.002	0.067	0.030	0.001	0.976	1.002	0.879–1.142
RDW-CV %	0.376	0.065	5.769	33.283	0.000	1.457	1.282–1.655
DBIL μmol/L	0.027	0.191	0.140	0.020	0.889	1.027	0.706–1.495
IBIL μmol/L	−0.005	0.104	−0.052	0.003	0.959	0.995	0.812–1.219
CysC mg/L	−0.012	0.718	−0.017	0.000	0.987	0.988	0.242–4.038
UA	−0.014	0.161	−0.088	0.008	0.930	0.986	0.720–1.351
Hcy μmol/L	−0.002	12.070	−0.000	0.000	1.000	0.998	0.000–7.605
SOD U/mL	0.162	0.075	4.686	0.030	0.586	1.176	0.015–1.361

MPAW, main pulmonary artery width; LAD, left atrial diameter; IVST, interventricular septal thickness; LVEDD, left ventricular end-diastolic diameter; RAD, right atrial diameter; RVD, right ventricular diameter; LVEF, left ventricular ejection fraction; NT-proBNP, N-terminal pro-brain natriuretic peptide; D-dimer, D-dimer (fibrin degradation product); WBC, white blood cell count; RBC, red blood cell count; HCT, hematocrit; RDW-CV, red cell distribution width; DBIL, direct bilirubin; IBIL, indirect bilirubin; UA, uric acid; CysC, cystatin C; Hcy, homocysteine; SOD, superoxide dismutase.

**Table 4:** Linear regression analysis results.

	Non-standardized coefficient		Standardization coefficient	*t*	*p*	Collinearity diagnostics	
	*B*	Standard error	Beta			VIF	Tolerance
Constant	−0.452	0.046	-	−9.745	0.000	-	-
Sex	−0.019	0.006	−0.025	−3.297	0.001	1.315	0.761
Age	0.002	0.000	0.053	6.921	0.000	1.298	0.771
Ethnicity	−0.007	0.003	−0.017	−2.523	0.012	1.030	0.971
Hypertension	−0.004	0.005	−0.005	−0.820	0.413	1.006	0.994
Type 2 diabetes	0.055	0.008	0.046	6.846	0.000	1.029	0.971
Smoking	0.070	0.008	0.065	8.793	0.000	1.230	0.813
MPAW mm	0.007	0.001	0.086	9.487	0.000	1.862	0.537
LAD mm	0.007	0.000	0.175	17.752	0.000	2.190	0.457
IVST mm	0.027	0.002	0.105	14.122	0.000	1.246	0.803
LVEDD mm	0.000	0.000	0.004	0.521	0.602	1.171	0.854
RAD mm	0.000	0.000	0.004	0.321	0.748	3.714	0.269
RVD mm	−0.006	0.001	−0.067	−6.891	0.000	2.129	0.470
LVEF%	−0.003	0.000	−0.075	−9.237	0.000	1.470	0.680
NT-pro BNP pg/mL	0.000	0.000	0.042	5.528	0.000	1.306	0.766
D-Dimer μg/mL	0.003	0.001	0.026	3.711	0.000	1.104	0.906
WBC × 10^9^/L	0.000	0.000	0.004	0.586	0.558	1.008	0.992
RBC × 10^9^/L	0.001	0.000	0.011	1.645	0.100	1.019	0.981
RDW-CV %	0.014	0.000	0.602	63.616	0.000	2.005	0.499
DBIL μmol/L	−0.000	0.000	−0.003	−0.327	0.743	1.832	0.546
IBIL μmol/L	−0.000	0.000	−0.007	−0.818	0.413	1.584	0.631
CysC mg/L	−0.008	0.002	−0.028	−4.133	0.000	1.051	0.952
UA μmol/L	−0.000	0.000	−0.111	−15.086	0.000	1.205	0.830
Hcy μmol/L	−0.000	0.000	−0.020	−2.946	0.003	1.022	0.978
SOD U/mL	0.001	0.000	0.059	7.637	0.000	1.322	0.756
D-W price	1.478						

Dependent variable = Discharge diagnosis. MPAW, main pulmonary artery width; LAD, left atrial diameter; IVST, interventricular septal thickness; LVEDD, left ventricular end-diastolic diameter; RAD, right atrial diameter; RVD, right ventricular diameter; LVEF, left ventricular ejection fraction; NT-proBNP, N-terminal pro-brain natriuretic peptide; D-dimer, D-dimer (fibrin degradation product); WBC, white blood cell count; RBC, red blood cell count; HCT, hematocrit; RDW-CV, red cell distribution width; DBIL, direct bilirubin; IBIL, indirect bilirubin; UA, uric acid; CysC, cystatin C; Hcy, homocysteine; SOD, superoxide dismutase.

#### 3.1.4 The predictive value of LAD, NT-proBNP, and RDW-CV for the presence of persistent AF

**Figure 1:**
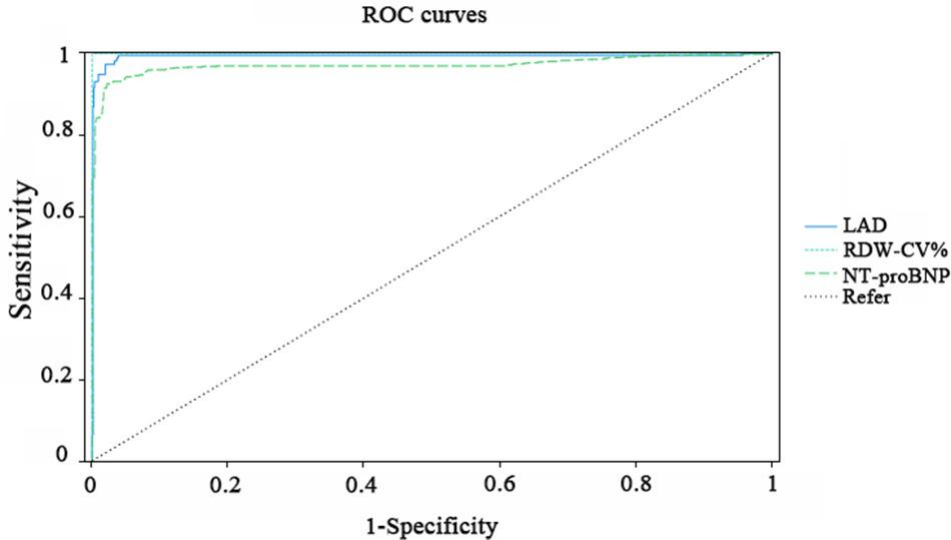
ROC curves of LAD, NT-proBNP, and RDW for predicting the presence of AF. ROC, receiver operating characteristic; LAD, left atrial diameter; NT-proBNP, N-terminal pro-brain natriuretic peptide; RDW-CV, red cell distribution width; AF, atrial fibrillation.

The ROC curve shows that the AUC value for LAD is 0.994, with a sensitivity of 0.994 and a specificity of 0.961. The AUC value for RDW is 0.996, with a sensitivity of 0.996 and a specificity of 0.976. The AUC value for NT-pro BNP is 0.971, with a sensitivity of 0.924 and a specificity of 0.978 (Figure 1).

### 3.2 Comparison of LAD, NT-proBNP, and RDW-CV in patients with AF from different ethnic groups in middle- and high-altitude areas

Patients of Hui ethnicity with AF demonstrated higher NT-proBNP, levels compared with those of Han and Tibetan ethnicities (*p* < 0.05). RDW-CV was lower among patients of Tibetan ethnicity compared with those of Han and Hui ethnicities (*p* < 0.05) as presented in Table 5.

**Table 5:** Comparative analysis of LAD, NT-proBNP, and RDW-CV among AF patients of different ethnic groups at middle and high altitudes.

Grouping	Indicator	Han Chinese (*n* = 1,094)	Tibetan (*n* = 143)	Hui (*n* = 200)	Others (*n* = 87)	*H*	*p*
Middle-altitude	LAD mm	66.000 (63.0, 66.0)	66.000 (65.0, 66.0)	66.000 (65.0, 66.0)	66.000 (63.0, 66.0)	1.048	0.790
	NT-proBNP pg/mL	2,393.000 (983.3, 4,962.8)	2,312.500 (775.7, 5,008.0)	3,613.000 (1,454.0, 6,541.0)	2,588.000 (976.0, 4,233.0)	24.623	0.000
	RDW-CV %	49.950 (45.9, 53.7)	49.200 (45.9, 53.1)	49.700 (46.6, 53.9)	48.550 (46.1, 51.6)	7.681	0.043
High-altitude	LAD mm	66.000 (64.0, 66.0)	66.000 (64.0, 66.0)	66.000 (63.0, 66.0)	66.000 (62.0, 66.0)	0.545	0.909
	NT-proBNP pg/mL	2,572.000 (958.0, 5,142.0)	2,511.500 (790.3, 4,675.3)	2,711.000 (1,145.5, 5,842.5)	2,502.000 (894.0, 5,145.8)	1.837	0.607
	RDW-CV %	49.400 (46.0, 53.6)	48.800 (45.9, 52.8)	50.300 (46.6, 56.2)	49.300 (45.7, 52.9)	8.304	0.040

Categorical variables are expressed as frequency (percentage). Continuous variables are expressed as median (25th percentile, 75th percentile) owing to non-normal distribution. AF, atrial fibrillation; LAD, left atrial diameter; NT-proBNP, N-terminal pro-brain natriuretic peptide; RDW-CV, red cell distribution width.

### 3.3 Comparison of LAD, NT-proBNP, and RDW-CV between patients with AF from Han, Tibetan, and Hui ethnic groups at middle and high altitudes

Patients with AF from the Hui ethnic group in middle-altitude areas had higher NT-proBNP levels compared to those in high-altitude areas (*p* < 0.05). However, there were no significant differences in LAD, NT-proBNP, and red blood cell distribution width levels among Han and Tibetan patients across different altitudes (*p* > 0.05), as presented in Table 6.

**Table 6:** Comparison of LAD, NT-proBNP, and RDW-CV between middle- and high-altitude regions among Han, Tibetan, and Hui patients with AF.

Ethnicity	Indicator	Middle-altitude (*n* = 1,945)	High-altitude (*n* = 1,094)	*Z*	*p*
Han	LAD mm	66.000 (63.0, 66.0)	66.000 (64.0, 66.0)	−0.073	0.942
	NT-proBNP pg/mL	2,393.000 (983.3, 4,962.8)	2,572.000 (958.0, 5,142.0)	−0.698	0.485
	RDW-CV %	49.200 (45.9, 53.1)	49.400 (46.0, 53.6)	−1.092	0.275
Tibetan	LAD mm	66.000 (65.0, 66.0)	66.000 (64.0, 66.0)	−0.300	0.764
	NT-proBNP pg/mL	2,312.500 (775.7, 5,008.0)	2,511.500 (790.3, 4,675.3)	−0.140	0.889
	RDW-CV %	49.650 (45.9, 53.7)	48.800 (45.9, 52.8)	−0.748	0.455
Hui	LAD mm	66.000 (65.0, 66.0)	66.000 (63.0, 66.0)	−0.992	0.321
	NT-proBNP pg/mL	3,613.000 (1,454.0, 6,541.0)	2,711.000 (1,145.5, 5,842.5)	−2.020	0.043
	RDW-CV %	49.700 (46.6, 53.9)	50.300 (46.6, 56.2)	−1.223	0.221

Categorical variables are expressed as frequency (percentage). Continuous variables are expressed as median (25th percentile, 75th percentile) owing to non-normal distribution. LAD, left atrial diameter; NT-proBNP, N-terminal pro-brain natriuretic peptide; RDW-CV, red cell distribution width.

## 4 Discussion

AF is a common arrhythmia with a multifactorial pathogenesis. LAD, NT-proBNP, and RDW-CV are independent factors associated with the presence of AF, and there are differences in NT-proBNP and RDW-CV levels among different ethnic groups.

The China Real-World Study on AF (RWS-CAF; registration number: ChiCTR1900021250) reported an AF prevalence of 2.3%, with prevalence increasing with age. Advanced age, male sex, and cardiovascular disease were identified as independent risk factors for AF (20).

In this study, multivariable analysis identified LAD, NT-proBNP, and RDW-CV as independent factors associated with AF. Previous echocardiographic evaluations by Gupta et al. demonstrated that approximately 55% of patients with AF exhibit left atrial enlargement and reduced left atrial ejection fraction (21). The current findings support these observations, indicating that increased LAD is predictive of AF. NT-proBNP, synthesized by atrial and ventricular myocytes, increases in response to cardiac overload or enlargement (22). AF-related contractile dysfunction elevates cardiac load and NT-proBNP levels, often along with left atrial enlargement (23). These results are consistent with the findings of Staszewsky et al., confirming NT-proBNP as a predictive biomarker for AF (24).

In addition, prior studies have indicated that RDW, specifically RDW-CV, contributes to AF development through mechanisms including inflammation, oxidative stress, and autonomic nervous system dysfunction (25, 26). This study corroborates these reports, establishing RDW-CV as an independent factor associated with AF.

Recent studies have found that RDW-CV can reflect the inflammatory and oxidative stress status of the body. Inflammation and oxidative stress are involved in the formation of AF, and inflammation appears to be a strong trigger of AF. Persistent AF appears to create and maintain an environment conducive to inflammation (27). Inflammatory infiltration, fibrosis, apoptosis, and even necrosis of myocardial cells lead to atrial enlargement and tissue fibrosis, promoting the presence of AF (28).

The presence of AF is closely associated with abnormalities in cardiac structure and function, with variations observed among patients of different ethnicities (29, 30). AF can reduce ventricular filling and impair systolic function, thereby adversely affecting diastolic performance. Recurrent AF episodes or excessively rapid ventricular rates may induce tachycardia-induced cardiomyopathy, with bidirectional interactions between these conditions. In this study, patients of Tibetan and Hui ethnicities with AF in both middle-altitude and high-altitude regions exhibited larger LVEDD compared with those of Han ethnicity, indicating a stronger association of AF on ventricular systolic function in these populations and potentially reflecting the higher prevalence of dilated cardiomyopathy among patients of Tibetan and Hui ethnicities (31). These ethnic differences may involve genetic factors, warranting further investigation.

LAD was confirmed as an independent risk factor for AF, exerting a consistent influence across ethnic groups. Patients of Hui ethnicity with AF residing in middle-altitude regions demonstrated higher NT-proBNP levels than other ethnic groups, which may be related to the greater prevalence of dilated cardiomyopathy in this population (32). Although the effect per unit increase is small, NT-proBNP shows substantial inter-individual variability in clinical settings, and the overall risk may therefore increase.

Oxidative stress and inflammatory markers, including RDW-CV, contribute to AF development and progression by affecting cardiac function (33, 34). Patients of Tibetan ethnicity with AF residing in middle-altitude and high-altitude regions had lower RDW-CV values compared to those of Hui and Han ethnicities, indicating a potentially greater role of inflammation in AF pathogenesis among patients of Han ethnicity.

Across altitudes, the factors associated with AF among ethnic groups were not entirely consistent, which may partly reflect the single-center design and potential biases of this study. In this study, altitude was not associated with the presence of AF. Comparisons of cardiac structure and erythrocyte metabolic parameters among patients of the same ethnicity residing at different altitudes demonstrated no significant differences between the middle-altitude and high-altitude regions. These findings indicate that, although altitude affects cardiac structure and RDW-CV, it does not alter the progression of AF. Future studies comparing plateau and lowland populations are warranted to clarify the impact of altitude on the presence of AF.

This study has several limitations. First, it was a single-center retrospective study, with all participants recruited from one hospital, which may limit the generalizability of the findings. Second, potential selection bias and the lack of matching in the control group may have introduced residual confounding that could not be fully eliminated despite regression adjustment. In addition, some variables showed relatively wide confidence intervals, which may be related to limited sample sizes in the corresponding subgroups. Future studies with larger sample sizes are needed for further validation. Third, the study population consisted exclusively of patients from middle- and high-altitude regions, without representation from low-altitude areas, which may limit the applicability of the results to other populations. Fourth, as a retrospective case-control study, the temporal sequence between LAD, RDW-CV, NT-proBNP, and AF cannot be established, and the possibility of reverse causation cannot be excluded. Future large-scale, multicenter, prospective studies are warranted to further validate these findings. Nevertheless, the present results reinforce LAD, NT-proBNP, and RDW-CV as important factors associated with the presence of AF and highlight differences observed across ethnic populations, providing a basis for further exploration of the pathogenesis of AF in diverse ethnic groups.

In conclusion, this study of patients with AF in Qinghai Province indicates that LAD, NT-proBNP, and RDW-CV are independent factors associated with AF.

## Data Availability

The raw data supporting the conclusions of this article will be made available by the authors, without undue reservation.
